# Rapid Acoustic Survey for Biodiversity Appraisal

**DOI:** 10.1371/journal.pone.0004065

**Published:** 2008-12-30

**Authors:** Jérôme Sueur, Sandrine Pavoine, Olivier Hamerlynck, Stéphanie Duvail

**Affiliations:** 1 Muséum national d'Histoire naturelle, Département Systématique et Évolution, UMR 5202 CNRS & USM 601 MNHN, CP 50, Paris, France; 2 Mathematical Ecology Research Group, Department of Zoology, University of Oxford, Oxford, United Kingdom; 3 Muséum national d'Histoire naturelle, Département Écologie et Gestion de la Biodiversité, UMR 5173 CNRS-UPMC & USM 305 MNHN, CP 51, Paris, France; 4 Centre for Ecology and Hydrology, Crowmarsh Gifford, Wallingford, Oxfordshire, United Kingdom; 5 IRD (Institut de Recherche pour le Développement), UR 200, IFRA, Nairobi, Kenya; University of Sussex, United Kingdom

## Abstract

Biodiversity assessment remains one of the most difficult challenges encountered by ecologists and conservation biologists. This task is becoming even more urgent with the current increase of habitat loss. Many methods–from rapid biodiversity assessments (RBA) to all-taxa biodiversity inventories (ATBI)–have been developed for decades to estimate local species richness. However, these methods are costly and invasive. Several animals–birds, mammals, amphibians, fishes and arthropods–produce sounds when moving, communicating or sensing their environment. Here we propose a new concept and method to describe biodiversity. We suggest to forego species or morphospecies identification used by ATBI and RBA respectively but rather to tackle the problem at another evolutionary unit, the community level. We also propose that a part of diversity can be estimated and compared through a rapid acoustic analysis of the sound produced by animal communities. We produced α and β diversity indexes that we first tested with 540 simulated acoustic communities. The α index, which measures acoustic entropy, shows a logarithmic correlation with the number of species within the acoustic community. The β index, which estimates both temporal and spectral dissimilarities, is linearly linked to the number of unshared species between acoustic communities. We then applied both indexes to two closely spaced Tanzanian dry lowland coastal forests. Indexes reveal for this small sample a lower acoustic diversity for the most disturbed forest and acoustic dissimilarities between the two forests suggest that degradation could have significantly decreased and modified community composition. Our results demonstrate for the first time that an indicator of biological diversity can be reliably obtained in a non-invasive way and with a limited sampling effort. This new approach may facilitate the appraisal of animal diversity at large spatial and temporal scales.

## Introduction

Biodiversity assessment is a central and urgent task in conservation biology, not only to determine species richness but also to evaluate differences between communities occupying different areas or changing with time [1]–[6]. The total species diversity in a set of communities has been traditionally seen as the product of the average diversity within communities (α) and the diversity between communities (β) [1]. There is a variety of methods of measuring α and β diversity. For example, the diversity between two communities can be calculated as the average change (turnover) in species composition between two communities [7]. Quantifying biological diversity mainly relies on species inventories that are both costly and challenging to compile [8]–[10]. The assessment of β diversity requires that the identities of species are known, which has prevented the analysis of β at broad spatial scales, especially when more than one taxon is considered [7]. This is particularly true for all-taxa biodiversity inventories (ATBI) which seek to identify every living species in an area and require several years of efforts and an important group of specialists [11]. Sampling brings major complications and, in most cases it is illusory to record and compare absolute species richness of communities in a short time. Numerous biodiversity indexes have therefore been invented to extrapolate from limited inventory data [12]. The consideration of the abundance of species especially led to biodiversity indexes less sensitive to sample size. These indexes, however, still require an important sampling effort to produce reliable estimates. Moreover, using such indexes demands a considerable sampling effort if diversity in a range of invertebrate and vertebrate animals needs to be assessed *e.g.* in high biodiversity tropical forests. Alternatively, one can focus on one taxon and assume that it predicts the diversity of other taxa. However, to claim that this taxon is a reliable indicator, several criteria regarding its biological properties have to be objectively tested, again involving a vast sampling effort in at least one 〈〈typical〉〉 location [13]. In addition, hotspots of species richness, for different taxa rarely coincide with the lowest correlation at finer spatial scales, which render difficult the definition of an indicator taxon or even combinations of several indicators supposedly representative of the diversity in other forms of organisms [14]. The mechanisms underlying such differences among taxa are still not understood [4]. A solution is to undertake rapid biodiversity assessment (RBA) as the Rapid Assessment Program (RAP) undertaken by Conservation international [15]. These programs rely on parataxonomists who only identify morphospecies or “recognizable taxonomic units” (RTU) [16]–[18]. This approach does not seem to be adequate for species inventories, population ecology or biogeography but can provide useful data for descriptions and global comparisons of species richness [19].

Another way to obtain a fast indicator of biodiversity and to allow inexpensive long-term, large-area monitoring of this indicator is to take advantage of an indirect cue of diversity. Several animals–birds, mammals, amphibians, fishes and arthropods–produce sounds incidentally when moving or intentionally when communicating or sensing their environment with sonar-like systems [20]. These organisms reveal their presence through acoustic signals that can be easily detected, recorded, saved and analysed. A first intuitive approach is to try to automatically identify assemblages of singing species like bats [21], birds [22], amphibians or insects [23]. However, automatic species identification has some limitations: it is sensitive to noise and it requires extensive preliminary study to establish templates for recognition processes, detailed acoustic analyses (*e.g.* dynamic time warping, cepstral coefficients, linear predictive coefficients, image processing) and complex computational methods (*e.g.* artificial neural network, hidden Markov model, Gaussian mixture model) [21]–[24]. Even if attractive, these methods have not yet been mainstreamed as a tool for obtaining a global measure of biodiversity. Because of their limitations, acoustics have occasionally been used to describe the temporal and spatial structure of tropical forest communities but very rarely to estimate local diversity. Using an array of microphones, spectral signatures have been defined for day, dusk and night times of a Bornean rainforest canopy [25] and two South American forests [26]. Reporting on the succession of different acoustic communities along the circadian cycle, these analyses did suggest that biodiversity could be monitored acoustically but, to our knowledge, a biodiversity index indirectly based on acoustic cues has only been computed in a single case where a classical Shannon-Wiener index [27] was based on the occurrence of twenty cricket species calling in an Amazonian rainforest [28]. Limited to a single insect taxon and preconditioned by a complete description of the signals produced by each cricket species, such an estimation is time consuming and difficult to repeat with other taxa or in other habitats.

Here we propose a new concept and method to describe biodiversity. We suggest to forego species or morphospecies identification used by ATBI and RBA respectively but rather to tackle the problem at another evolutionary unit, the community level. We first make the simple assumption that the more species are found in a community the more different signals will be produced at the same time. This will increase the heterogeneity of the acoustic environment. In addition, species singing in the same area and at the same time face the risk of mutual masking interference [29]. Acoustic space is a single resource that has to be shared by competitive singing species. As such, signals should show species-specific frequency and temporal patterns that minimize the effects of overlap from other species [30]. This leads to a partitioning of both sender and receiver acoustic space as reported in several assemblages [28], [31]–[37]. By over-dispersion of temporal and frequency parameters, partitioning should then also increase acoustic space heterogeneity. This effect should be even more significant for stable communities than for perturbed communities where recent invader species might have changed the acoustic equilibrium of the community [32]. Using simple signal analysis, we developed new α and β diversity indexes based on the analysis of the acoustic choruses. We tested both indexes with simulated animal communities and applied them two Tanzanian forests within the hotspot of the Eastern Arc and the Coastal Forests of Tanzania and Kenya [38].

## Materials and Methods

### Acoustic Entropy Index (H)

If *x*(*t*) is a time series of length *n*, the amplitude envelope of oscillation is obtained with the analytic signal *ξ*(*t*) of *x*(*t*). The analytic signal is defined as:
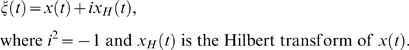
(1)The probability mass function of the amplitude envelope *A*(*t*) is obtained as:
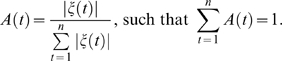
(2)In signal theory [27], the entropy *H* of a random variable *X* with probability mass function *p_X_*(*x*) is defined as [39]:
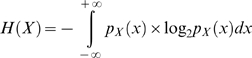
(3)


Shannon index is the second most used index of diversity in ecology, after species richness (number of species) [40]. In general, it is measured on a set of categories differing in frequencies. It increases with the evenness of the frequencies of the categories and with the number of categories. In ecology, categories are often species that differ by their relative abundances in a community. Here we apply it on a time series sequence of size *n*, the categories are the time units and their frequencies are the probability mass function of the amplitude envelope. The prevalence of Shannon index over other indices especially the Simpson index has a long history of debates [12]. Its main characteristic is that it is more sensitive to rare categories [41]. Therefore by using this index, the time units with low probability mass function of the amplitude envelope will still influence the value of the acoustic diversity. The maximum value of Shannon index depends on the number of categories (log_2_(*n*)). The sounds of animals in field will affect the amplitude envelope at each time unit. However the number of time units is fixed by the methodology. Consequently, to obtain an index that is solely affected by the sounds of animals in field, we divide the Shannon index by its maximum. The index obtained measures the evenness of the amplitude envelope over the time units.

The temporal entropy *H_t_* is then computed following:
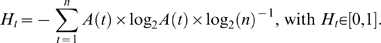
(4)Similarly, to calculate the spectral entropy, a mean spectrum *s*(*f*) is first computed using a Short Time Fourier Transform (STFT) based on a non-overlapping sliding function window of sample width *τ*. This mean spectrum *s*(*f*) is similarly transformed into a probability mass function *S(f)* of length *N* used to compute the spectral entropy *H_f_*:
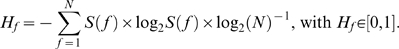
(5)Eventually, the entropy index *H* is computed as the product of both temporal and spectral entropies (*H* = *H_t_*×*H_f_*, with 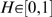). *H* will tend towards 0 for a single pure tone, increases with the number of frequency bands and amplitude modulations, and tends towards 1 for a random noise. We tested the hypothesis that *H* index increases with the number of singing species. To achieve this, we simulated series of choruses composed of different numbers of species. Thirty seconds recordings (16 bit digitization, 44.1 kHz sampling frequency) of 45 common singing species of the western Palaearctic region were first obtained from professional recordings [42]–[44] and personal recordings (J. Sueur). These 45 species included 15 avian, 15 amphibian and 15 insect species (Table S1). All these species can potentially be found singing in close proximity. These 45 recordings were randomly divided in three groups of 15 species with 5 avian, 5 amphibian and 5 insect species each. To generate a series of choruses, ten species recordings were first randomly taken within a group. Recordings' amplitudes originally matched but a relative amplitude level varying from 0.1 to 1 by step of 0.1 was then randomly affected to each recording. This allowed getting different amplitudes for each species, a necessary condition to mimic natural conditions. The recordings were then successively and randomly added leading to ten different sound files, starting with a sound file with a single species to a sound file with ten distinct species. This procedure was repeated 10 times for each group leading to 300 sound files (3 groups×10 series×10 choruses). *H_t_* was computed with envelopes lasting 30 s (*n* = 1 323·10^6^ points). The frequency precision of the STFT was 83.13 Hz (*τ* = 512 samples). The resulting mean spectrum used to compute *H_f_* was made of *N* = 256 elements. *H* index could be then calculated for each of the 300 chorus generated (Fig. S1 and Tables S1, S2).

### Acoustic Dissimilarity Index (D)

We extended a measure estimating the compositional dissimilarity between two communities [45] to both envelope and spectral acoustic data. Envelope dissimilarity between two signals *x_1_(t)* and *x_2_(t)* of the same duration digitized at the same sampling frequency can be estimated by computing the difference between their envelope probability mass functions divided by 2 to get values between 0 and 1:
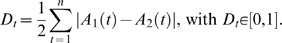
(6)Similarly, spectral dissimilarity can be assed by computing:
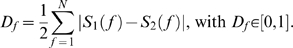
(7)


The dissimilarity acoustic index is computed as the product of both temporal and spectral dissimilarities (*D* = *D_t_*×*D_f_*, with 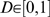). We tested the hypothesis that *D* index increases with the number of unshared species between chorus pairs. We simulated a new set of choruses based on the same three groups of 15 species each as for the application of the acoustic entropy index. In each group, we first randomly chose seven recordings among the 15 available. Thus seven amplitude-weighted recordings were added giving the first chorus of the series. From this starting chorus, we randomly replaced one of the species recordings by a new one randomly chosen from the eight remaining species recordings. We then obtained two choruses differing in a single species. We repeated this species recording swap eight successive times knowing that a replaced species could not be replaced a second time according to a random choice without replacement. This process led to eight distinct choruses differing from one to seven species. All this procedure was repeated ten times generating ten series of eight choruses for each group. Consequently, we obtain a total of 240 sound files (3 groups×10 series×8 choruses). Among each series, the *D* index was computed between the first and the successive choruses with similar STFT parameters used when calculating *H* index (Fig. S1, Tables S1, S3).

### Tanzanian Coastal Forests

Sound recordings were achieved in two Tanzanian coastal forests located in the Rufiji valley (Rufiji District) and distant of 50 km. The two dry lowland coastal forests studied are characterized by different degrees of degradation [46]. Ngumburuni forest, north of the Rufiji River, has been exploited since German colonial times, especially for iroko trees (*Milicia exelsa*) used in joinery, shipbuilding, civil engineering (Fig. S2a). The second forest, Kichi Hills, is situated south of the Rufiji River and has been hard to access until the completion of a bridge in 2003 [47] (Fig. S2b). Prior to 1992 it was very selectively logged for large *Milicia exelsa* but it has not been exploited since. A recording spot was randomly chosen inside each forest avoiding any edge effect that could affect species richness (Ngumburuni: UTM 37M 505351–9128198, 41 m altitude; Kichi Hills: UTM 37L 462443–9088710, 575 m alt). Recordings were done by a single observer in 2007 from the 4^th^ to the 9^th^ April in Ngumburuni (spot 1) and from 9^th^ to the 14^th^ April in Kichi (spot 2). In both forests the dawn-dusk choruses, known to be the noisiest periods of the day in tropical forests [48], were recorded within a ten day period (4 to 14 April 2007) with 5 consecutive days spent in each forest. They were made at three day times corresponding to the highest acoustic activity period in the forests: (1) dawn chorus from 6.00 am to 6.15 am, (2) first dusk chorus from 5.30 pm to 5.45 pm and (3) second dusk chorus from 6.30 pm to 6.45 pm. This resulted in 30 recording sessions (2 sites×5 days×3 day times) for a total of 450 minutes. One recording in Kichi had to be withdrawn from the analysis because of a heavy rain generating important noise. In all cases, weather conditions were assessed during each recording session by measuring the ambient temperature (±1°C) and the relative humidity (±0.5%). This was completed by relative indexes referring to discrete meteorological scales. The scale describing cloud cover was: (0) no clouds, (1) 1–50% cloud cover, (2) 50–75% cloud cover, (3) 100% cloud cover, and (4) rain. The scale reporting wind force was: (0) no wind, (1) leaves motion, (2) leaves and branches motion, (3) leaves, branches and trunks motion. The recording equipment consisted in an omni-directional Sennheiser K6/ME62 microphone (frequency response: ±2.5 dB between 0.02 and 20 kHz) connected to an Edirol R-09 digital recorder (16 bit digitization at 44.1 kHz sampling frequency). The recording level was similarly set up for all recording sessions. The microphone was always held vertically by hand at a height of 2 m. Before processing entropy and dissimilarity analyses, a 170 Hz high-pass filter was applied to all sound files. This selectively removed the lowest frequencies due to wind noise only. *H_t_* was computed with envelopes lasting 900 s ( = 3 969·10^7^ points). The frequency precision of the STFT was 83.13 Hz. The resulting mean spectrum used to compute *H_f_* was made of 256 elements. *H* index was then computed for each recording session. *D* index was estimated for every pair of recording sessions.

All statistics were computed using *R* [49]. *H* and *D* indexes were computed by writing specific *R* functions specifically implemented in the free package *seewave* [50].

## Results

### Development and test of α and β acoustic indexes

**Figure 1:**
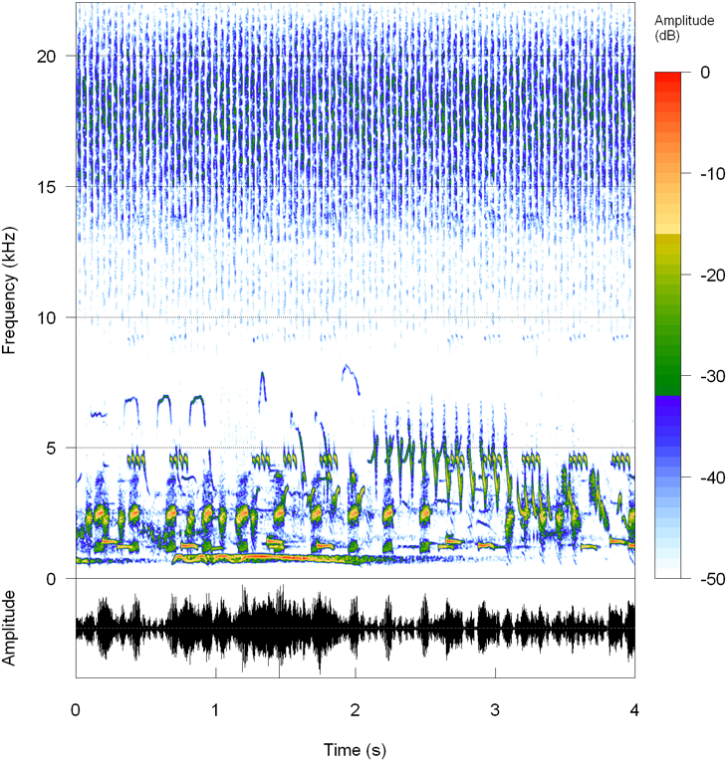
Example of a random simulated chorus. Waveform and spectrogram showing frequency profile over time, amplitude being depicted with a relative decibels (dB) colour scale. The chorus, which originally lasted 30 s, includes 5 birds (*Fringilla coelebs*, *Parus major*, *Strix aluco*, *Troglodytes troglodytes*, *Turdus merula*), 5 amphibians (*Alytes obstetricans*, *Bufo bufo*, *Hyla arborea*, *Pelodytes punctatus*, *Rana ridibunda*), and 5 insects (*Chrysocraon dispar*, *Cicada orni*, *Gryllus campestris*, *Metrioptera bicolor*, *Oecanthus pellucens*).

**Figure 2:**
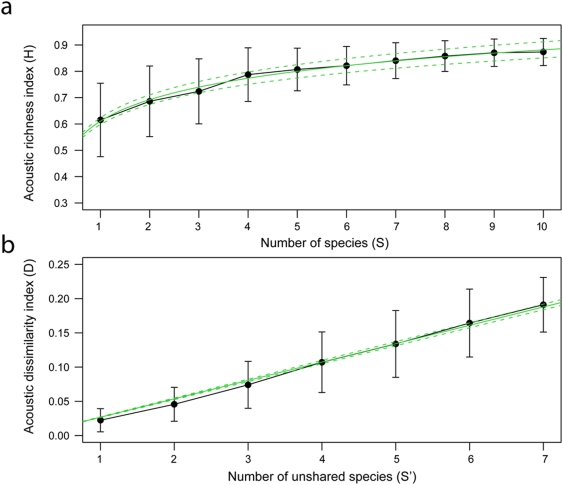
Acoustic entropy *H* index and dissimilarity *D* index tested with simulated choruses. (*a*) evolution of *H* index in relation with the number of species composing the chorus. *H* was calculated for eight chorus series among which the number of species per chorus varied from one to 10. Non-linear regression: *H* = 0.1176×log(*S*)+0,6107, *n* = 300. (*b*) evolution of *D* index in relation with the number of unshared species between choruses. *D* was calculated for eight chorus series including seven species each among which the number of species differed from one (14.3%) to seven (100%). Linear-regression with null intercept: *D* = 0,0268×*S*′, *F* = 2054, *R^2^* = 0.908, *n* = 240. Error bars indicate standard deviation. Regressions are plotted with solid lines and their 95% confidence intervals with dotted lines.

*H* values ranged between 0.369 and 0.948 and increased with species richness *S* following a logarithmic model (Fig. 1, Fig. 2a, Audio S1). Heterogeneity of the sound emitted by the community is then positively linked to the number of species within the community. As shown by standard deviation which decreases from 0.140 for single species choruses to 0.051 for choruses including ten species, the variability of *H* decreases with *S*. *D* index is null for similar signals and tends towards 1 for completely different signals. To test *D*, we randomly generated 240 choruses differing in the number of species they share. We used the same 45 species sample when testing *H*. We found that, in average, *D* increases linearly from 0.022±0.017 (*n* = 30 pairs of simulated choruses differing by a single species) to 0.191±0.040 (*n* = 30 pairs of simulated choruses differing by all the seven species they include) with the number of unshared species between pairs of choruses (Fig. 2b).

### Application of the acoustic indexes to two African coastal forests

**Figure 3:**
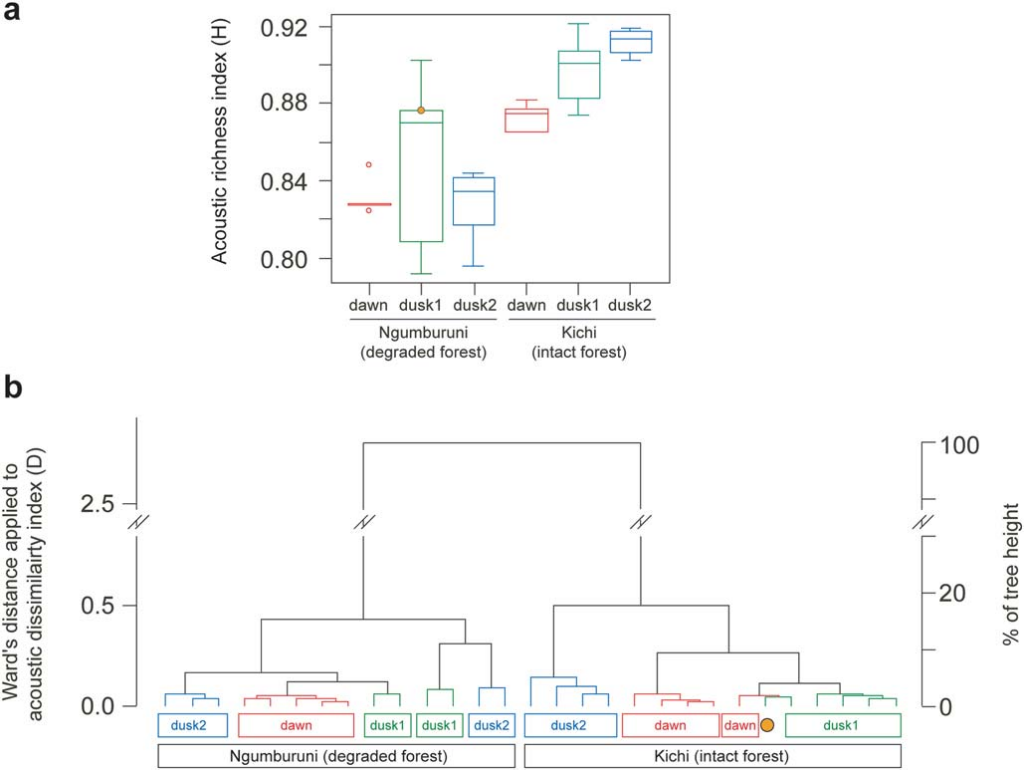
Acoustic richness and dissimilarity of two Tanzanian lowland coastal forests. (*a*) Variation of *H* within and among forests. (*b*) Ward's hierarchical cluster analysis applied to *D* index. The orange dot refers to the single misclassified recording between sites. “Dawn” = chorus from 6.00 am to 6.15 am, “dusk1” = chorus from 5.30 pm to 5.45 pm, “dusk2” = chorus from 6.30 pm to 6.45 pm. Boxes are bounded by the first quartile, median, and third quartile; whiskers are 1.5 times the interquartile range; points outside the whiskers are outliers.

Ngumburuni degraded forest appeared to be warmer (Kruskal-Wallis *χ^2^* = 14.102, *P* = 0.002), dryer (Kruskal-Wallis *χ^2^* = 17.784, *P* = 2.475·10^−5^), less windy (Kruskal-Wallis *χ^2^* = 7.174, *P* = 0.007) and less cloudy (Kruskal-Wallis *χ^2^* = 9.804, *P* = 0.002) than Kichi intact forest (table S4). These meteorological differences may have had some effect on animal activity. However, temperature, which is the main factor regulating acoustic behaviour of exothermal animals, ranged in an interval (25.09°C±2.66, min = 21.7°C, max = 31°C) where sound production is not constrained [51]–[52]. We computed *H* index for each forest at three times of the day (dawn chorus, and two dusk choruses). We found that *H* values were significantly higher for the intact forest (*H* = 0.891±0.023, *n* = 14) than for the degraded forest (*H* = 0.836±0.030, *n* = 15) (Fig. 3a; Kruskal-Wallis *χ^2^* = 15.420, *df* = 1, *P* = 8.57·10^−5^) suggesting a higher diversity. Furthermore, we found increasing *H* values with low variance from dawn to dusk in the intact forest (Kruskal-Wallis *χ^2^* = 7.790, *df* = 2, *P* = 0.020) but not in the degraded forest (Kruskal-Wallis *χ^2^* = 0.560, *df* = 2, *P* = 0.756).

**Figure 4:**
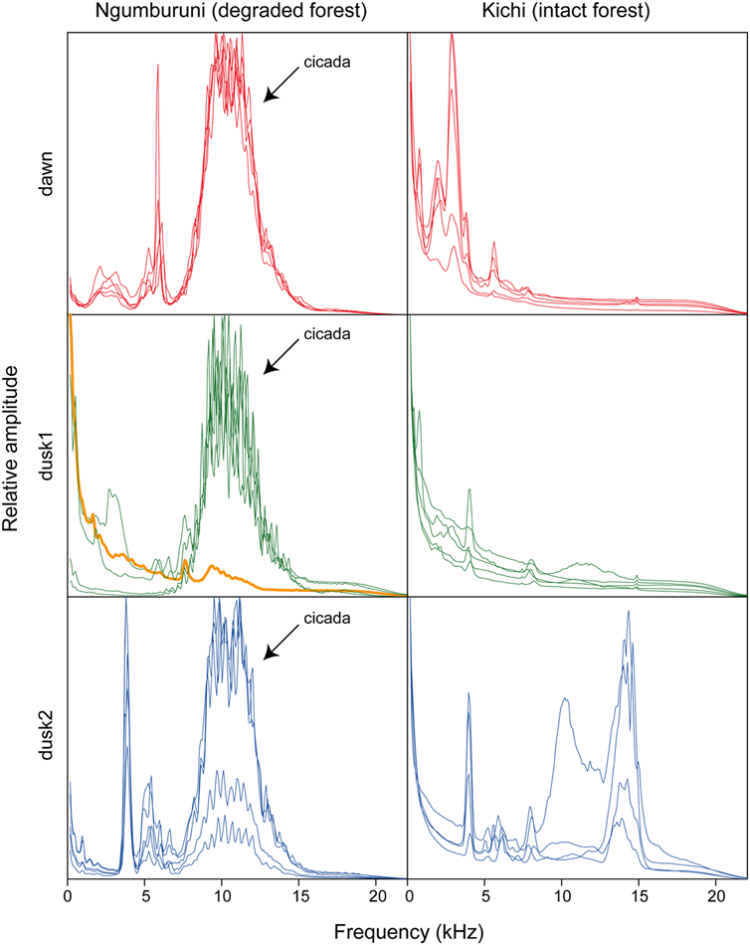
Spectral profiles of two Tanzanian lowland coastal forests. Mean spectral profiles of the two forests at tree times of the day. The plots depict variations of amplitude (sound energy) over frequencies. Each line corresponds to one recording session. Arrow indicates the cicada species singing in Ngumburuni. The orange profile refers to the recording misclassified (Fig. 3). “Dawn”, “dusk1” and “dusk2” refers to three recording times along the day, see Fig. 3 for details.

We then applied *D* index between every recording session pair. *D* index essentially reveals a difference between the two forests (distance-based redundancy analysis [53] with 1,000 permutations, *df* = 1, *F* = 46.730, *P*<0.001). A Ward's hierarchical cluster analysis correctly classified all recordings except one according to sites (Fig. 3b). In addition, *D* exhibits differences in the course of the day that are obvious in the intact forest (distance-based redundancy analysis [53] with 1,000 permutations, *df* = 2, *F* = 11.100, *P*<0.001) but less strong in the degraded one even if significant (distance-based redundancy analysis [53] with 1,000 permutations, *df* = 2, *F* = 3.270, *P*<0.001, Fig. 3a). One recording made in the degraded forest is misclassified in the intact forest (Fig. 3b). This dusk recording is distinguished by the absence of acoustic activity in the range of 7–15 kHz usually occupied by one cicada species (Fig. 4, Figs. S3, S4, S5, S6, S7, S8 and Audio S2, S3, S4, S5, S6, S7).

## Discussion

Global biodiversity assessment at large spatial and temporal scales needs fast and reliable methods to rapidly assess and compare species richness in both accessible and remote habitats. Taking advantage of the sound produced by active animals, our objective was to build diversity indexes easy to compute and repeat. Tested with artificial choruses for which the number of species is known, we have shown that an α diversity index, *H*, derived from the Shannon information statistic [27] increases from 0 to 1 with signal entropy, or heterogeneity. Higher values of *H* would then indicate richer habitats. The variability of *H* decreases with the number of species indicating that some error can be expected for communities with very few species. This can occur, for instance, when a single species produces a sound covering a broad spectrum of frequencies. This was particularly the case with cicada species that emit noise-like songs and where seasonality might introduce a bias. Similarly, some noise due to wind, running water, or human activities could reduce the reliability of the *H* index. However, as achieved with the recordings made in Tanzania, a high-pass filter with a cut-off frequency around 200 Hz can be used to selectively remove the low frequency components due to noise only. To help conservation planners in their decisions, it is necessary to compare areas in order to locate the centers of maximal diversity and above all the temporal changes in the diversity of a region. We designed a β diversity index, *D*, based on surface differences between envelopes and spectral content of the signals to be compared. When applying *D* index between pairs of artificial communities, results clearly show a linear increase of *D* values with the number of unshared species. Even if we were not able to estimate the upper threshold of *D* values, these tests clearly indicate that *D* could be used to infer differences between community compositions.

All these preliminary tests were achieved with random simulated choruses that have not been shaped by selective forces. We then applied our method to two dry lowland coastal forests of Tanzania which rank eighth on the biodiversity hotspot list [38]. These forests are of primary interest due to high concentrations of endemic species and are threatened by conversion to agriculture, charcoal production and logging [54]. Species inventories have already been undertaken but there is still a dramatic need for biodiversity measurement and mapping [55]. The two dry lowland forests are characterized by different degrees of degradation mainly due to an historical natural barrier to human impact until 2003 [46]–[47]. We found that *H* values were significantly higher for the intact forest than for the degraded one and they increased with low variance from dawn to dusk in the intact forest but not in the degraded forest. The *D* index clearly highlights differences between the two forests leading to a high-level in their acoustic classification (Fig. 3). This index also reveals important differences between the three periods of the day for the intact forest. These results first suggest a higher diversity in the intact forest. This would also indicate the existence of at least three acoustic communities with few overlap between species songs in the intact forest whilst there might be only a single community in the degraded forest with more acoustic interference between species. Degradation might have then changed both composition and time activity pattern of the Ngumburuni communities. The degraded Ngumburuni forest was probably occupied by communities with overlapping compositions during the day. This was mainly due to the presence of a cicada species at the three periods of the day. The peak due to this cicada species was the most striking difference between the two forests at 5.30–5.45 pm (dusk1) as shown by the frequency spectrum of the degraded forest when the cicada species was exceptionally absent (Fig. 4). However, this was not the only source of difference. At 6.00–6.15 am (dawn) the peaks at low frequency in the intact forest are absent from the degraded forest. At 6.30–6.45 pm (dusk2), other peaks at high frequencies characterized the intact forest only. Our diversity index *H* measures the evenness of the acoustic space. Consequently, if a few species dominate the acoustic space, then the diversity will be low. More abundant species might then notably reduce the acoustic diversity of a habitat, as it reduces classical index of diversity such as Simpson and Shannon indexes. Furthermore, if few species are widespread and dominate in an area, then the differences between local communities (*D* index) will be low even if secondary species make sounds at different frequencies but low amplitude. As forest degradation is known to change communities' composition [10], the ecological success of the cicada species may have contributed to a decrease in diversity. The highest diversity detected in the intact forest seems to be due to a higher number of species and to equilibrium in their relative abundance. The spectral profiles show indeed a higher dispersion of amplitude peaks along the frequency axis, suggesting that species share the available acoustic space more equitably. The following hypothesis can be drawn from our results: the intact forest would be close to an acoustic stable state while the degraded forest would have moved away from this acoustic homeostasis.

Even if our method need to be tested over larger samples in nature and for habitats which the fauna has been previously listed, we have shown that as more species occupy the same habitat the soundscape they generate is more heterogeneous. We have also shown that differences between acoustic communities could be evaluated through simple signal analysis. As our method does not require specific skills, biodiversity estimation through acoustics can be undertaken by non-scientists. This will, eventually, allow monitoring at large spatial and temporal scales, opening up new opportunities in biodiversity research.

## Supporting Information

Figure S1 Protocol principle followed to simulate choruses used when testing H and D indexes. See text for details.
(0.23 MB PNG)

Figure S2 Trails in both Tanzanian forests where recording were achieved: (a) the degraded Ngumburuni forest, (b) the intact Kichi Hills forest.
(1.01 MB PNG)

Figure S3 Sample of a dawn chorus [6.00–6.15 am] recorded in the degraded Ngumburuni forest. Waveform and spectrogram showing frequency profile over time, amplitude being shown with a relative decibels (dB) colour scale. 7th April 2007, 24.5°C, 81% h.r. 170 Hz high-pass filtered to remove noise due to wind. See Sound S2.
(0.56 MB PNG)

Figure S4 Sample of a first dusk chorus [5.30–5.45 pm] recorded in the degraded Ngumburuni forest. Waveform and spectrogram showing frequency profile over time, amplitude being shown with a relative decibels (dB) colour scale. 7th April 2007, 28.9°C, 74% h.r. 170 Hz high-pass filtered to remove noise due to wind. See Sound S3.
(0.55 MB PNG)

Figure S5 Sample of a second dawn chorus [6.30–6.45 pm] recorded in the degraded Ngumburuni forest. Waveform and spectrogram showing frequency profile over time, amplitude being shown with a relative decibels (dB) colour scale. 6th April 2007, 28°C, 84% h.r. See Fig. S4. 170 Hz high-pass filtered to remove noise due to wind. See Sound S4.
(0.19 MB PNG)

Figure S6 Sample of a dawn chorus [6.00–6.15 am] recorded in the intact Kichi Hills forest. Waveform and spectrogram showing frequency profile over time, amplitude being shown with a relative decibels (dB) colour scale. 10th April 2007, 22.8°C, 94% h.r. Hz high-pass filtered to remove noise due to wind. See Sound S2.
(0.38 MB PNG)

Figure S7 Sample of a first dusk chorus [5.30–5.45 pm] recorded in the intact Kichi Hills forest. Waveform and spectrogram showing frequency profile over time, amplitude being shown with a relative decibels (dB) colour scale. 9th April 2007, 25.6°C, 81% h.r. 170 Hz high-pass filtered to remove noise due to wind. See Sound S3.
(0.56 MB PNG)

Figure S8 Sample of a second dawn chorus [6.30–6.45 pm] recorded in the intact Kichi Hills forest. Waveform and spectrogram showing frequency profile over time, amplitude being shown with a relative decibels (dB) colour scale. 12th April 2007, 23.5°C, 93% h.r. 170 Hz high-pass filtered to remove noise due to wind. See Sound S4.
(0.75 MB PNG)

Table S1 List of the 45 species recordings used when testing H and D indexes. They were randomly divided in three groups of five birds, five amphibians and five insects each.
(0.05 MB DOC)

Table S2 Reference chorus series used when testing the H test. Ten series of ten choruses were generated with the recordings listed and coded in Table S1.
(0.04 MB DOC)

Table S3 Reference chorus series used when testing the D index. Ten series of eight choruses were generated with the recordings listed and coded in Table S1.
(0.06 MB DOC)

Table S4 Local meteorological conditions during recording sessions in the two Tanzanian lowland coastal forests. Results are given as mean±sd (sample size).
(0.03 MB DOC)

Audio S1 Example of a simulated chorus. The chorus, which originally lasts 30 s, includes 5 birds (Fringilla coelebs, Parus major, Strix aluco, Troglodytes troglodytes, Turdus merula), 5 amphibians (Alytes obstetricans, Bufo bufo, Hyla arborea, Pelodytes punctatus, Rana ridibunda), and 5 insects (Chrysocraon dispar, Cicada orni, Gryllus campestris, Metrioptera bicolor, Oecanthus pellucens). See Fig. 1.
(0.07 MB MP3)

Audio S2 81% h.r. 170 Hz high-pass filtered to remove noise due to wind. See Fig. S3.
(0.24 MB MP3)

Audio S3 Sample of a first dusk chorus in the degraded Ngumburuni forest. 7th April 2007, 28.9°C, 74% h.r. 170 Hz high-pass filtered to remove noise due to wind. See Fig. S4.
(0.24 MB MP3)

Audio S4 Sample of a second dusk chorus in the degraded Ngumburuni forest. 6th April 2007, 28°C, 84% h.r. 170 Hz high-pass filtered to remove noise due to wind. See Fig. S5.
(0.24 MB MP3)

Audio S5 Sample of a dawn chorus in the intact Kichi Hills forest. 10th April 2007, 22.8°C, 94% h.r. 170 Hz high-pass filtered to remove noise due to wind. See Fig. S6.
(0.24 MB MP3)

Audio S6 Sample of a first dusk chorus in the intact Kichi Hills forest. 9th April 2007, 25.6°C, 81% h.r. 170 Hz high-pass filtered to remove noise due to wind. See Fig. S7.
(0.24 MB MP3)

Audio S7 Sample of a second dusk chorus in the intact Kichi Hills forest. 12th April 2007, 23.5°C, 93% h.r. 170 Hz high-pass filtered to remove noise due to wind. See Fig. S8.
(0.24 MB MP3)
