# Supplementary figures and images for: Rapid Acoustic Survey for Biodiversity Appraisal
Source: PLoS One. 2008 Dec 30;3(12):e4065. doi: 10.1371/journal.pone.0004065 (PMC2605254; doi:10.1371/journal.pone.0004065)

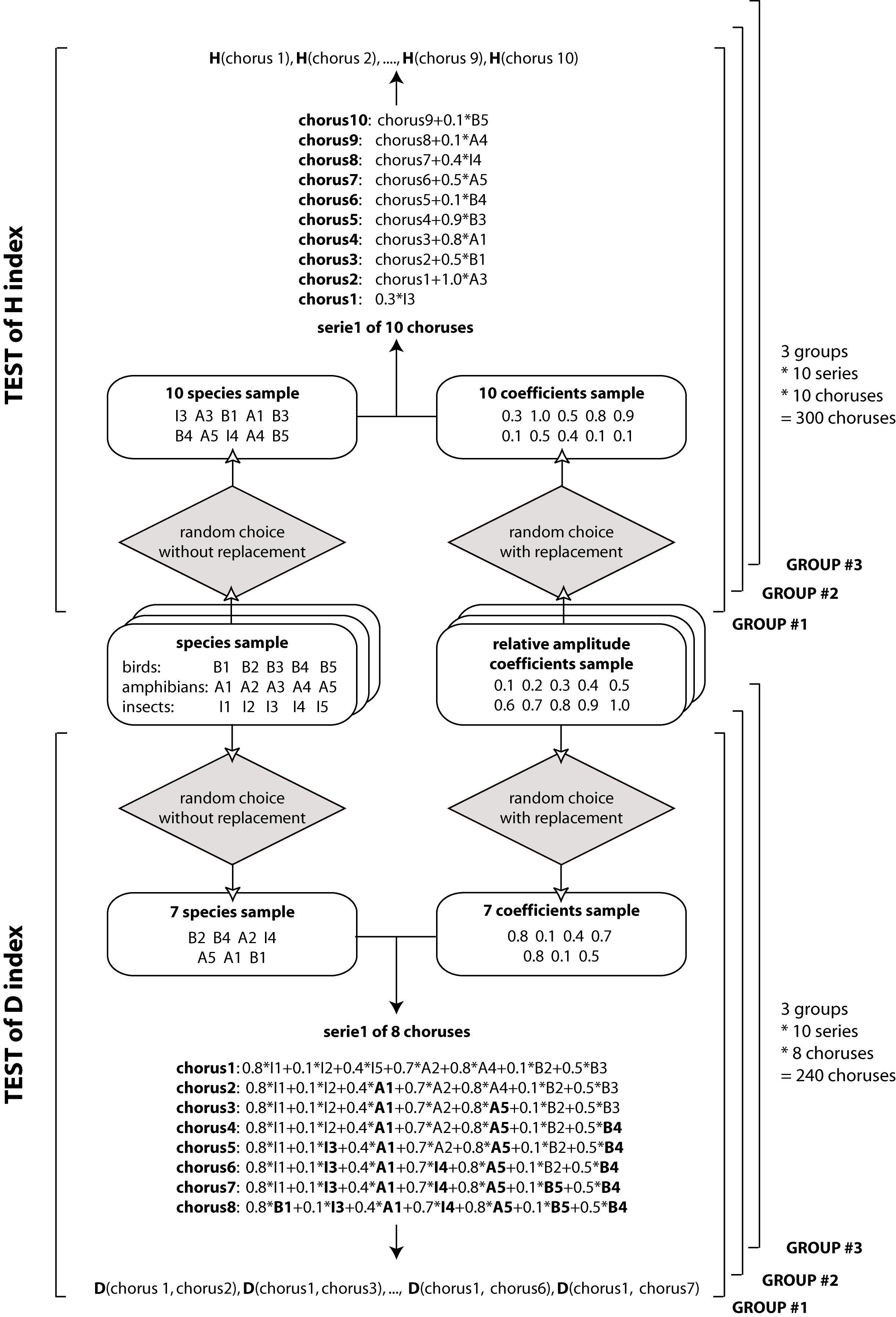

Supplement: Figure S1 — Protocol principle followed to simulate choruses used when testing H and D indexes. See text for details. (0.23 MB PNG) [file pone.0004065.s001.png]

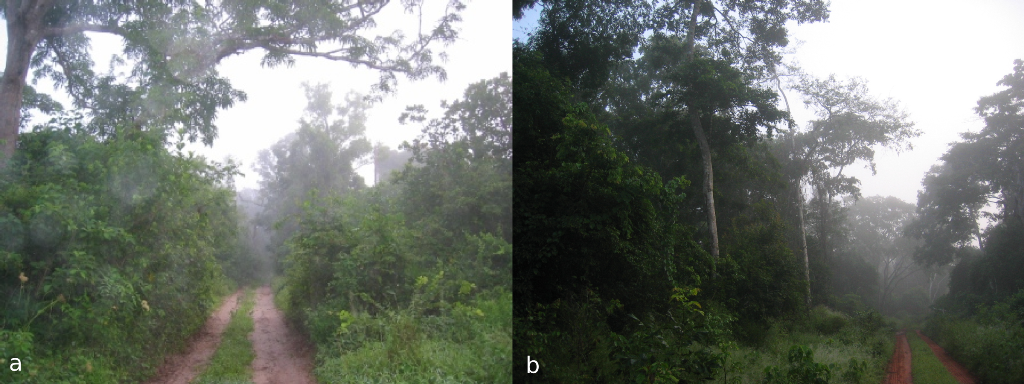

Supplement: Figure S2 — Trails in both Tanzanian forests where recording were achieved: (a) the degraded Ngumburuni forest, (b) the intact Kichi Hills forest. (1.01 MB PNG) [file pone.0004065.s002.png]

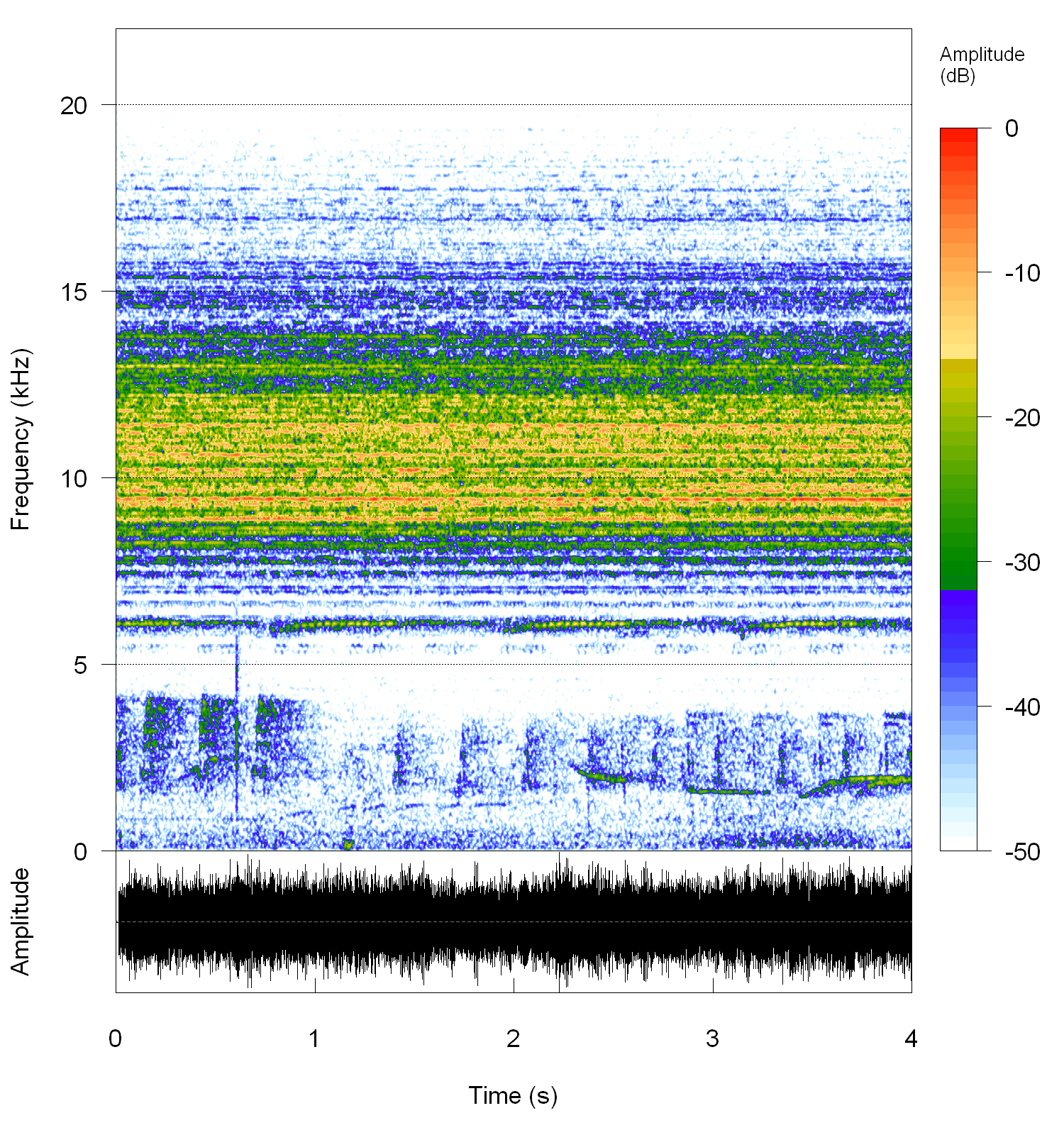

Supplement: Figure S3 — Sample of a dawn chorus [6.00–6.15 am] recorded in the degraded Ngumburuni forest. Waveform and spectrogram showing frequency profile over time, amplitude being shown with a relative decibels (dB) colour scale. 7th April 2007, 24.5°C, 81% h.r. 170 Hz high-pass filtered to remove noise due to wind. See Sound S2. (0.56 MB PNG) [file pone.0004065.s003.png]

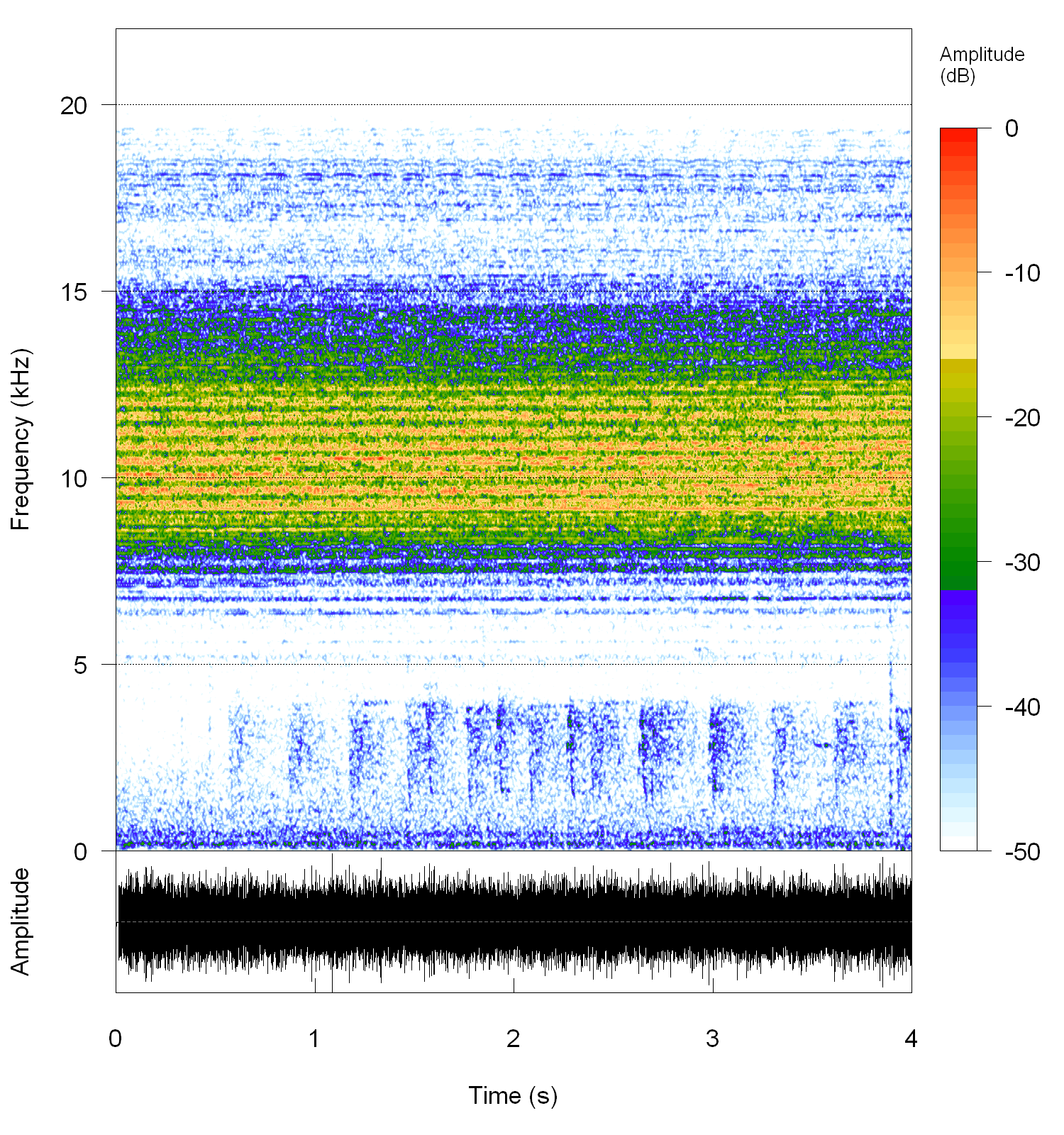

Supplement: Figure S4 — Sample of a first dusk chorus [5.30–5.45 pm] recorded in the degraded Ngumburuni forest. Waveform and spectrogram showing frequency profile over time, amplitude being shown with a relative decibels (dB) colour scale. 7th April 2007, 28.9°C, 74% h.r. 170 Hz high-pass filtered to remove noise due to wind. See Sound S3. (0.55 MB PNG) [file pone.0004065.s004.png]

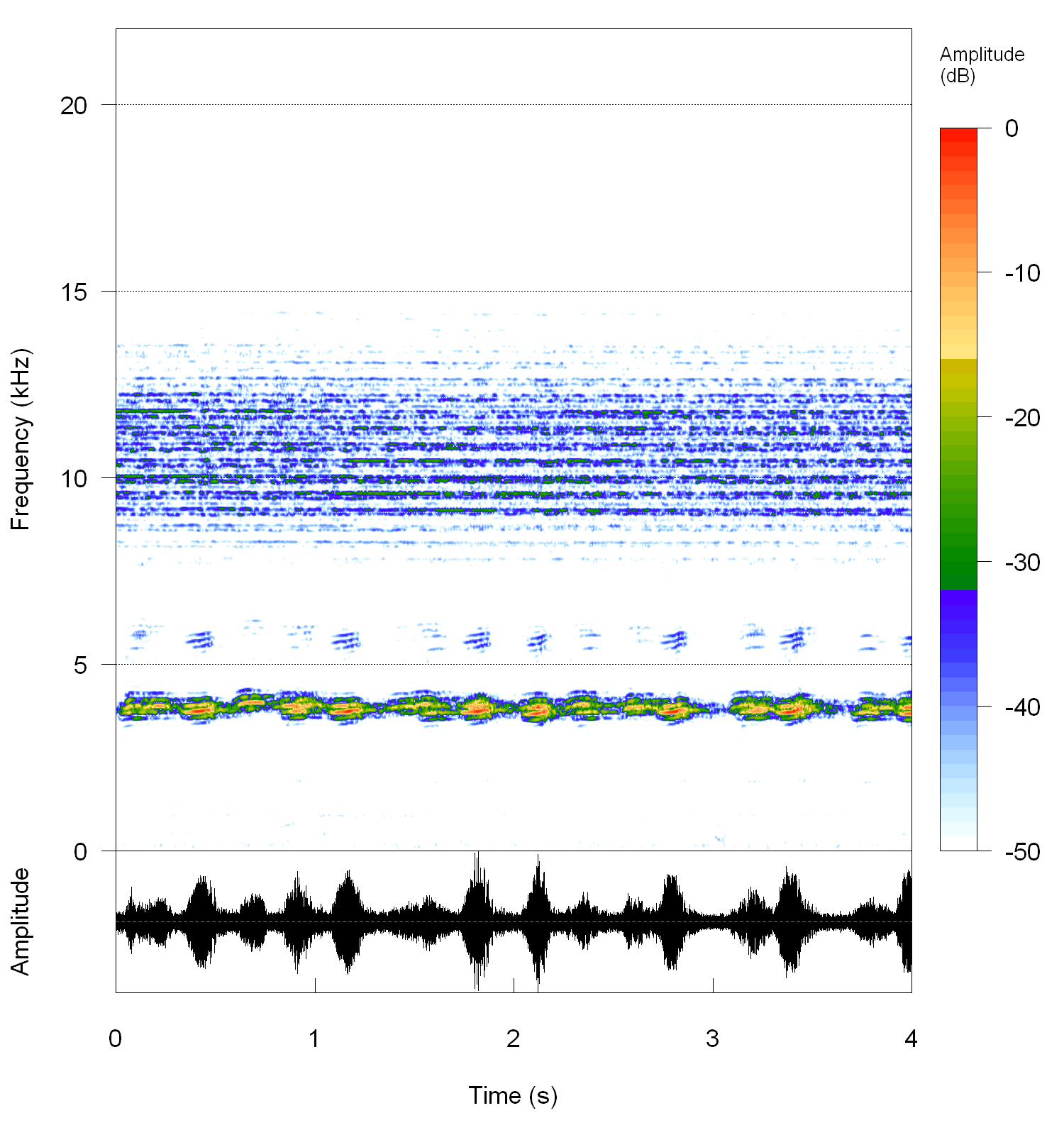

Supplement: Figure S5 — Sample of a second dawn chorus [6.30–6.45 pm] recorded in the degraded Ngumburuni forest. Waveform and spectrogram showing frequency profile over time, amplitude being shown with a relative decibels (dB) colour scale. 6th April 2007, 28°C, 84% h.r. See Fig. S4. 170 Hz high-pass filtered to remove noise due to wind. See Sound S4. (0.19 MB PNG) [file pone.0004065.s005.png]

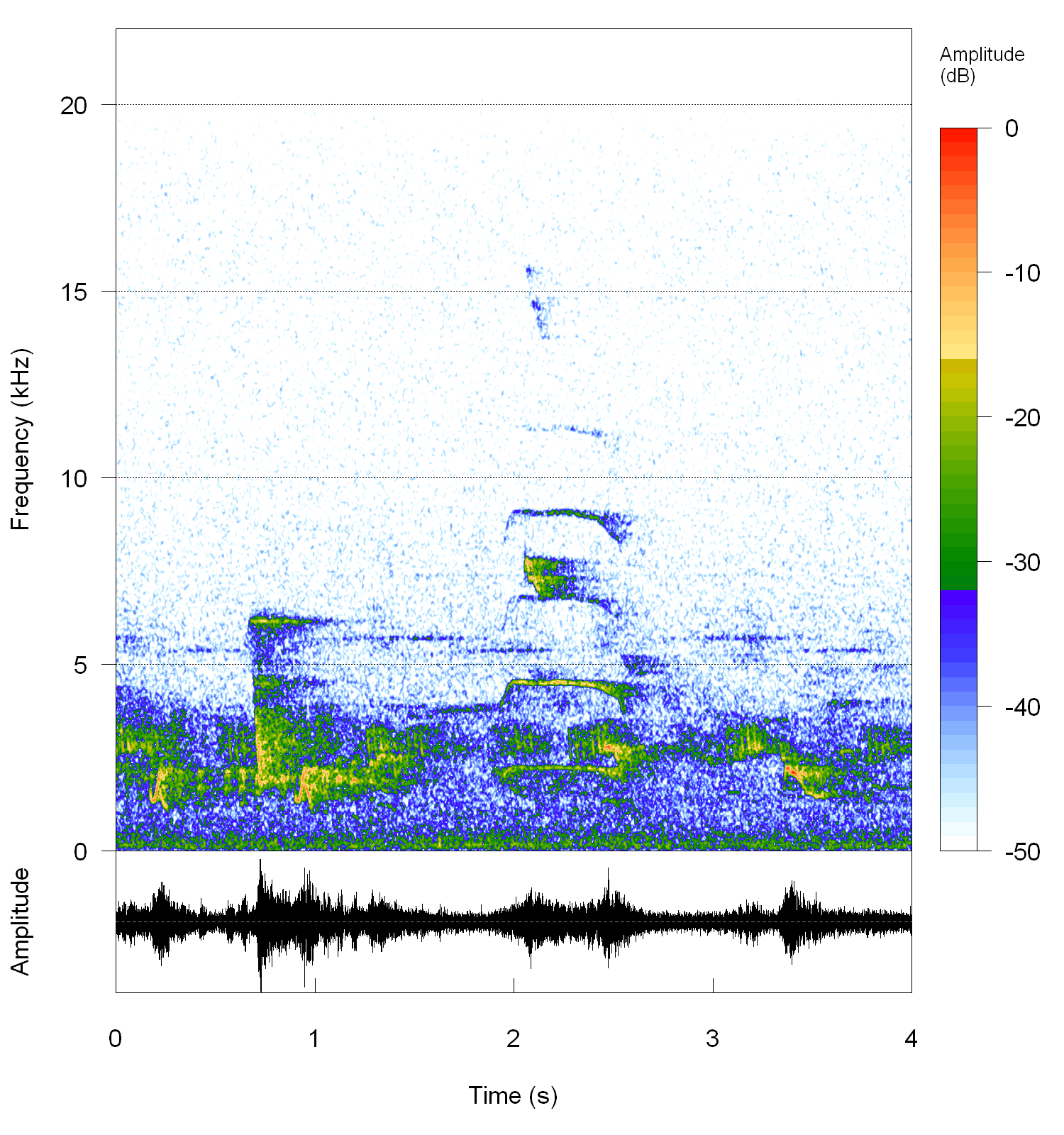

Supplement: Figure S6 — Sample of a dawn chorus [6.00–6.15 am] recorded in the intact Kichi Hills forest. Waveform and spectrogram showing frequency profile over time, amplitude being shown with a relative decibels (dB) colour scale. 10th April 2007, 22.8°C, 94% h.r. Hz high-pass filtered to remove noise due to wind. See Sound S2. (0.38 MB PNG) [file pone.0004065.s006.png]

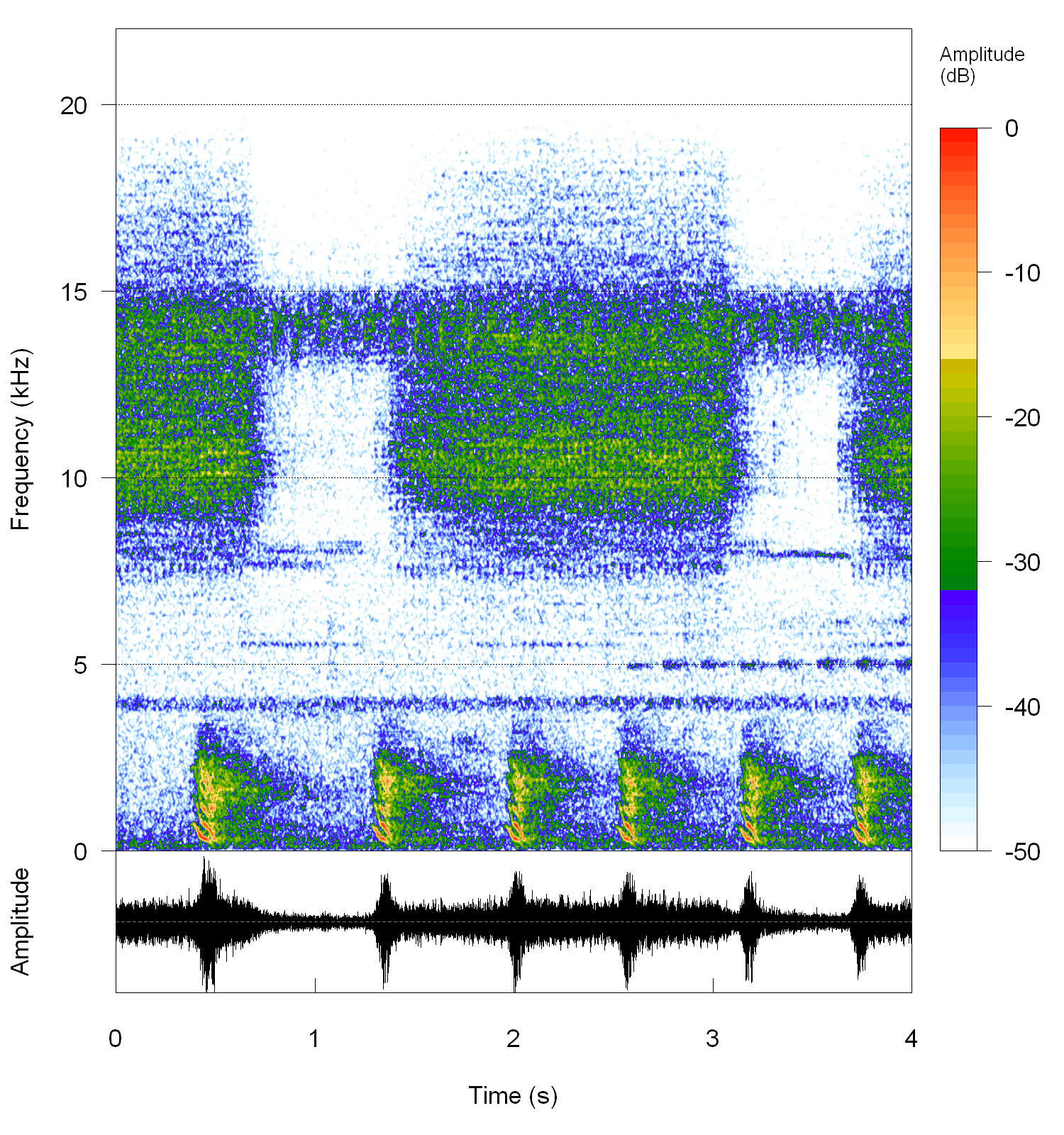

Supplement: Figure S7 — Sample of a first dusk chorus [5.30–5.45 pm] recorded in the intact Kichi Hills forest. Waveform and spectrogram showing frequency profile over time, amplitude being shown with a relative decibels (dB) colour scale. 9th April 2007, 25.6°C, 81% h.r. 170 Hz high-pass filtered to remove noise due to wind. See Sound S3. (0.56 MB PNG) [file pone.0004065.s007.png]

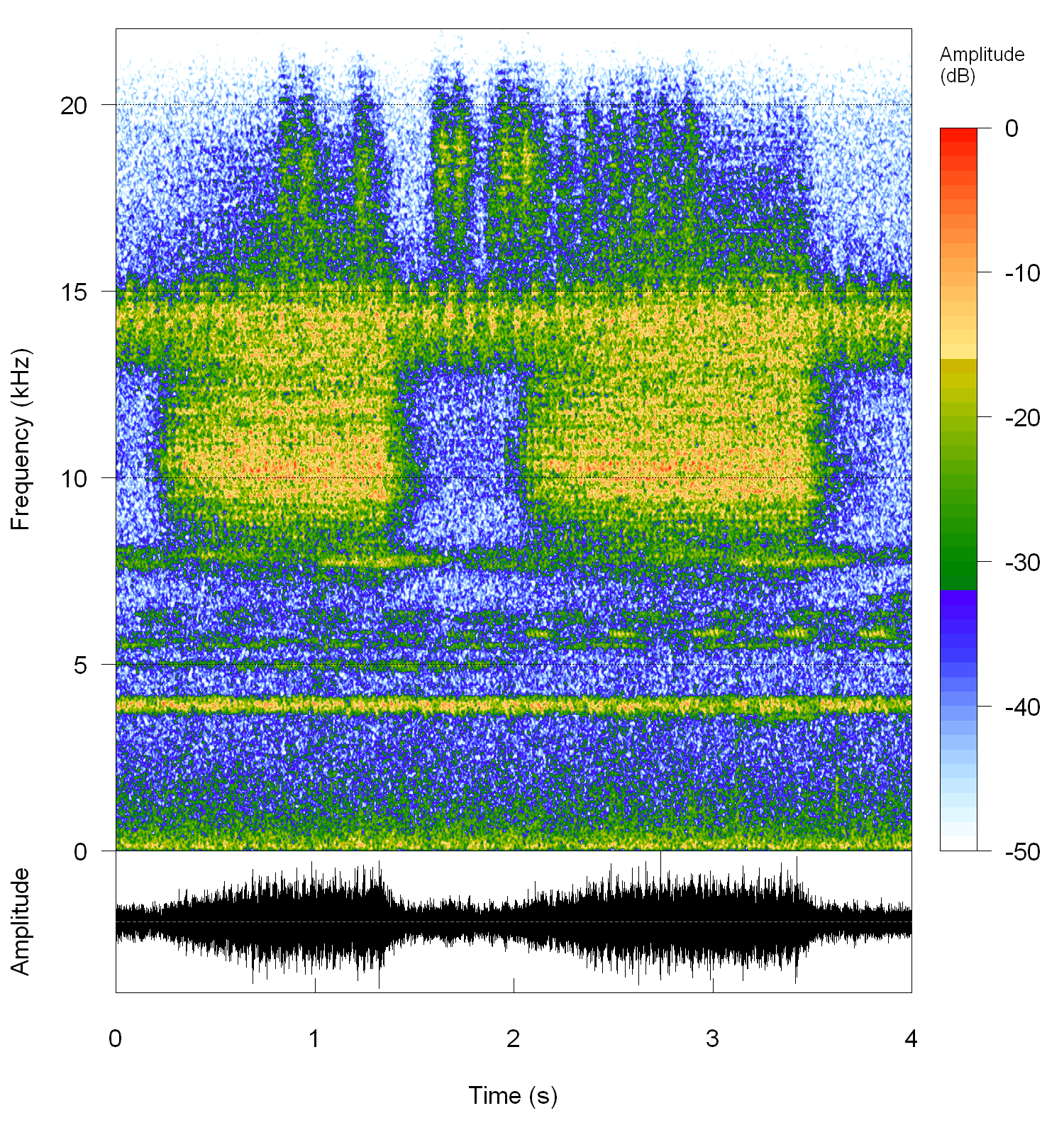

Supplement: Figure S8 — Sample of a second dawn chorus [6.30–6.45 pm] recorded in the intact Kichi Hills forest. Waveform and spectrogram showing frequency profile over time, amplitude being shown with a relative decibels (dB) colour scale. 12th April 2007, 23.5°C, 93% h.r. 170 Hz high-pass filtered to remove noise due to wind. See Sound S4. (0.75 MB PNG) [file pone.0004065.s008.png]
