# Supplementary material for: Rapid Acoustic Survey for Biodiversity Appraisal
Source: PLoS One. 2008 Dec 30;3(12):e4065. doi: 10.1371/journal.pone.0004065 (PMC2605254; doi:10.1371/journal.pone.0004065)
Supplement: Table S1 — List of the 45 species recordings used when testing H and D indexes. They were randomly divided in three groups of five birds, five amphibians and five insects each. (0.05 MB DOC) [file pone.0004065.s009.doc]

**Table S1.** List of the 45 species recordings used when testing *H* and *D* indexes. They were randomly divided in three groups of five birds, five amphibians and five insects each.

|  |  | **GROUP 1** | | **GROUP 2** | | **GROUP 3** | |
| --- | --- | --- | --- | --- | --- | --- | --- |
| **Order** | **Code** | **Species** | **Recording source** | **Species** | **Recording source** | **Species** | **Recording source** |
| Bird | B1 | *Fringilla coelebs* | Deroussen (2001) [1] | *Prunella modularis* | Deroussen (2001) [1] | *Sturnus vulgaris* | Deroussen (2001)[1] |
| Bird | B2 | *Parus major* | Deroussen (2001) [1] | *Sylvia atricapilla* | Deroussen (2001) [1] | *Turdus philomelos* | Deroussen (2001)[1] |
| Bird | B3 | *Strix aluco* | Deroussen (2001) [1] | *Passer domesticus* | Deroussen (2001) [1] | *Phylloscopus trochilus* | Deroussen (2001)[1] |
| Bird | B4 | *Troglodytes troglodytes* | Deroussen (2001) [1] | *Alauda arvensis* | Deroussen (2001) [1] | *Pica pica* | Deroussen (2001)[1] |
| Bird | B5 | *Turdus merula* | Deroussen (2001) [1] | *Streptopelia turtur* | Deroussen (2001)[1] | *Serinus serinus* | Deroussen (2001)[1] |
| Amphibian | A1 | *Alytes obstetricans* | Deroussen *et al*. (2003)[2] | *Hyla meridionalis* | Deroussen *et al*. (2003)[2] | *Rana temporaria* | Deroussen *et al*. (2003)[2] |
| Amphibian | A2 | *Bufo bufo* | Deroussen *et al*. (2003)[2] | *Rana dalmatina* | Deroussen *et al*. (2003)[2] | *Bufo calamita* | Deroussen *et al*. (2003)[2] |
| Amphibian | A3 | *Hyla arborea* | Deroussen *et al*. (2003)[2] | *Bufo viridis* | Deroussen *et al*. (2003)[2] | *Pelobates fuscus* | Deroussen *et al*. (2003)[2] |
| Amphibian | A4 | *Pelodytes punctatus* | Deroussen *et al*. (2003)[2] | *Rana ridibunda* | Deroussen *et al*. (2003)[2] | *Bombina variegata* | Deroussen *et al*. (2003)[2] |
| Amphibian | A5 | *Rana ridibunda* | Deroussen *et al*. (2003)[2] | *Rana lessonae* | Deroussen *et al*. (2003)[2] | *Rana arvalis* | Deroussen *et al*. (2003)[2] |
| Insect | I1 | *Chrysocraon dispar* | J. Sueur | *Lyristes plebejus* | J. Sueur | *Tibicina haematodes* | J. Sueur |
| Insect | I2 | *Cicada orni* | J. Sueur | *Ephippiger ephippiger* | Bonnet (1995)[3] | *Decticus albifrons* | Bonnet (1995)[3] |
| Insect | I3 | *Gryllus campestris* | Bonnet (1995)[3] | *Gryllus bimaculatus* | Bonnet (1995)[3] | *Conocephalus discolor* | Bonnet (1995)[3] |
| Insect | I4 | *Metrioptera bicolour* | J. Sueur | *Chorthippus biguttulus* | Bonnet (1995)[3] | *Tartarogryllus bordigalensis* | Bonnet (1995)[3] |
| Insect | I5 | *Oecanthus pellucens* | J. Sueur | *Stenobothrus lineatus* | Bonnet (1995)[3] | *Chorthippus brunneus* | Bonnet (1995)[3] |

1. Deroussen F (2001) *Oiseaux des jardins de France* (Nashvert Production, Ligue de Protection des Oiseaux, 1CD, Charenton).

2. Deroussen F, Jollivet B, Fradet V, Goatmeur J, Faucheux P, Roché JC, Vences M, Chevalier J, Nöllert A, Serfling C*, et al.* (2003) *Guide sonore des Amphibiens de France, Belgique et Luxembourg* (Nashvert Production, Parthénope Collection, Charenton).

3. Bonnet FR (1995) *Guide sonore des sauterelles, grillons et criquets d'Europe occidentale* (Delachaux & Niestlé, Lausanne, Paris).
