# Supplementary material for: Rapid Acoustic Survey for Biodiversity Appraisal
Source: PLoS One. 2008 Dec 30;3(12):e4065. doi: 10.1371/journal.pone.0004065 (PMC2605254; doi:10.1371/journal.pone.0004065)
Supplement: Table S2 — Reference chorus series used when testing the H test. Ten series of ten choruses were generated with the recordings listed and coded in Table S1. (0.04 MB DOC) [file pone.0004065.s010.doc]

**Table S2.** Reference chorus series used when testing the *H* test. Ten series of ten choruses were generated with the recordings listed and coded in Table S1.

| **Chorus** | **Serie 1** | **Serie 2** | **Serie 3** | **Serie 4** | **Serie 5** | **Serie 6** | **Serie 7** | **Serie 8** | **Serie 9** | **Serie 10** |
| --- | --- | --- | --- | --- | --- | --- | --- | --- | --- | --- |
| **C1** | 0.3*I3 | 0.9*A4 | 0.5*I1 | 0.8*B3 | 0.1*B3 | 0.2*B2 | 0.5*B3 | 0.2*B4 | 0.7*I2 | 0.6*B3 |
| **C2** | C1+1.0*A3 | C1+0.7*A5 | C1+1.0*A5 | C1+0.7*A3 | C1+0.1*A4 | C1+0.6*A1 | C1+0.4*A1 | C1+0.8*I2 | C1+0.6*B4 | C1+0.6*I4 |
| **C3** | C2+0.5*B1 | C2+0.1*I2 | C2+1.0*I4 | C2+0.5*B4 | C2+0.3*A5 | C2+0.2*A5 | C2+0.1*A4 | C2+0.9*A2 | C2+0.7*B1 | C2+0.6*I1 |
| **C4** | C3+0.8*A1 | C3+0.6*A2 | C3+0.6*B2 | C3+0.5*I5 | C3+0.8*I1 | C3+0.5*B3 | C3+1.0*I5 | C3+1.0*A4 | C3+0.2*B5 | C3+1.0*B4 |
| **C5** | C4+0.9*B3 | C4+1.0*B3 | C4+0.3*A4 | C4+0.1*B2 | C4+0.9*B2 | C4+0.6*B1 | C4+0.7*B2 | C4+0.6*I3 | C4+0.7*I5 | C4+0.8*A2 |
| **C6** | C5+0.1*B4 | C5+0.1*I3 | C5+0.4*B1 | C5+0.1*B5 | C5+0.6*A3 | C5+0.3*I1 | C5+0.3*A3 | C5+1.0*A1 | C5+0.4*A3 | C5+0.9*I5 |
| **C7** | C6+0.5*A5 | C6+0.5*I4 | C6+1.0*I2 | C6+0.7*A4 | C6+0.3*B1 | C6+0.3*I4 | C6+0.3*I1 | C6+0.7*B3 | C6+0.7*A2 | C6+0.3*B1 |
| **C8** | C7+0.4*I4 | C7+0.5*A3 | C7+0.3*A2 | C7+0.3*A5 | C7+0.7*B5 | C7+0.4*I2 | C7+0.1*B4 | C7+0.6*I1 | C7+0.2*A5 | C7+0.6*A4 |
| **C9** | C8+0.1*A4 | C8+0.3*B5 | C8+1.0*A1 | C8+0.1*I4 | C8+0.5*I4 | C8+0.2*A3 | C8+0.3*I3 | C8+0.8*B2 | C8+0.7*I3 | C8+0.1*I3 |
| **C10** | C9+0.1*B5 | C9+0.9*I1 | C9+0.3*B5 | C9+1.0*A1 | C9+0.1*A2 | C9+1.0*I3 | C9+0.9*A2 | C9+0.2*A5 | C9+0.1*B3 | C9+0.3*B2 |
