# Supplementary material for: Rapid Acoustic Survey for Biodiversity Appraisal
Source: PLoS One. 2008 Dec 30;3(12):e4065. doi: 10.1371/journal.pone.0004065 (PMC2605254; doi:10.1371/journal.pone.0004065)
Supplement: Table S3 — Reference chorus series used when testing the D index. Ten series of eight choruses were generated with the recordings listed and coded in Table S1. (0.06 MB DOC) [file pone.0004065.s011.doc]

**Table S3.** Reference chorus series used when testing the *D* index. Ten series of eight choruses were generated with the recordings listed and coded in Table S1.

| **Chorus** | **Serie 1** | **Serie 2** |
| --- | --- | --- |
| **C1** | 0.8*I1+0.1*I2+0.4*I5+0.7*A2+0.8*A4+0.1*B2+0.5*B3 | 0.9*I2+0.9*I4+0.4*I5+0.9*A3+0.1*A4+0.9*B2+0.5*B4 |
| **C2** | 0.8*I1+0.1*I2+0.4*A1+0.7*A2+0.8*A4+0.1*B2+0.5*B3 | 0.9*I2+0.9*I4+0.4*I5+0.9*A2+0.1*A4+0.9*B2+0.5*B4 |
| **C3** | 0.8*I1+0.1*I2+0.4*A1+0.7*A2+0.8*A5+0.1*B2+0.5*B3 | 0.9*I2+0.9*I3+0.4*I5+0.9*A2+0.1*A4+0.9*B2+0.5*B4 |
| **C4** | 0.8*I1+0.1*I2+0.4*A1+0.7*A2+0.8*A5+0.1*B2+0.5*B4 | 0.9*I2+0.9*I3+0.4*I5+0.9*A2+0.1*A4+0.9*B2+0.5*B1 |
| **C5** | 0.8*I1+0.1*I3+0.4*A1+0.7*A2+0.8*A5+0.1*B2+0.5*B4 | 0.9*I1+0.9*I3+0.4*I5+0.9*A2+0.1*A4+0.9*B2+0.5*B1 |
| **C6** | 0.8*I1+0.1*I3+0.4*A1+0.7*I4+0.8*A5+0.1*B2+0.5*B4 | 0.9*I1+0.9*I3+0.4*I5+0.9*A2+0.1*A5+0.9*B2+0.5*B1 |
| **C7** | 0.8*I1+0.1*I3+0.4*A1+0.7*I4+0.8*A5+0.1*B5+0.5*B4 | 0.9*I1+0.9*I3+0.4*I5+0.9*A2+0.1*A5+0.9*B5+0.5*B1 |
| **C8** | 0.8*B1+0.1*I3+0.4*A1+0.7*I4+0.8*A5+0.1*B5+0.5*B4 | 0.9*I1+0.9*I3+0.4*B3+0.9*A2+0.1*A5+0.9*B5+0.5*B1 |
|  | **Serie 3** | **Serie 4** |
| **C1** | 1.0*I2+0.8*I3+0.1*I4+0.2*A1+0.9*A3+0.7*A5+0.4*B4 | 0.9*I1+0.1*I2+0.3*I4+0.5*I5+0.8*A5+0.4*B1+1.0*B2 |
| **C2** | 1.0*I2+0.8*A2+0.1*I4+0.2*A1+0.9*A3+0.7*A5+0.4*B4 | 0.9*I1+0.1*I2+0.3*I4+0.5*I5+0.8*A5+0.4*B1+1.0*B4 |
| **C3** | 1.0*I2+0.8*A2+0.1*I4+0.2*A1+0.9*A3+0.7*B2+0.4*B4 | 0.9*I1+0.1*I2+0.3*I4+0.5*A3+0.8*A5+0.4*B1+1.0*B4 |
| **C4** | 1.0*I2+0.8*A2+0.1*I4+0.2*I5+0.9*A3+0.7*B2+0.4*B4 | 0.9*I3+0.1*I2+0.3*I4+0.5*A3+0.8*A5+0.4*B1+1.0*B4 |
| **C5** | 1.0*B1+0.8*A2+0.1*I4+0.2*I5+0.9*A3+0.7*B2+0.4*B4 | 0.9*I3+0.1*I2+0.3*I4+0.5*A3+0.8*A1+0.4*B1+1.0*B4 |
| **C6** | 1.0*B1+0.8*A2+0.1*I4+0.2*I5+0.9*A3+0.7*B2+0.4*A4 | 0.9*I3+0.1*I2+0.3*B5+0.5*A3+0.8*A1+0.4*B1+1.0*B4 |
| **C7** | 1.0*B1+0.8*A2+0.1*I4+0.2*I5+0.9*I1+0.7*B2+0.4*A4 | 0.9*I3+0.1*A2+0.3*B5+0.5*A3+0.8*A1+0.4*B1+1.0*B4 |
| **C8** | 1.0*B1+0.8*A2+0.1*B3+0.2*I5+0.9*I1+0.7*B2+0.4*A4 | 0.9*I3+0.1*A2+0.3*B5+0.5*A3+0.8*A1+0.4*B3+1.0*B4 |
|  | **Serie 5** | **Serie 6** |
| **C1** | 0.2*I1+0.7*I5+0.7*A2+07*A3+0.9*A4+0.5*B3+0.4*B4 | 0.8*I3+0.9*I5+0.6*A1+0.4*A3+0.4*A4+0.6*A5+0.2*B4 |
| **C2** | 0.2*I1+0.7*I5+0.7*A2+0.7*A3+0.9*I3+0.5*B3+0.4*B4 | 0.8*I3+0.9*I5+0.6*A1+0.4*I4+0.4*A4+0.6*A5+0.2*B4 |
| **C3** | 0.2*I1+0.7*I5+0.7*A2+0.7*A3+0.9*I3+0.5*B3+0.4*B2 | 0.8*I3+0.9*I5+0.6*A1+0.4*I4+0.4*A5+0.6*B2+0.2*B4 |
| **C4** | 0.2*I1+0.7*I5+0.7*B1+0.7*A3+0.9*I3+0.5*B3+0.4*B2 | 0.8*I3+0.9*I5+0.6*A2+0.4*I4+0.4*A5+0.6*B2+0.2*B4 |
| **C5** | 0.2*I1+0.7*I4+0.7*B1+0.7*A3+0.9*I3+0.5*B3+0.4*B2 | 0.8*I3+0.9*I5+0.6*A2+0.4*I4+0.4*A5+0.6*B2+0.2*I2 |
| **C6** | 0.2*I1+0.7*I4+0.7*B1+0.7*A1+0.9*I3+0.5*B3+0.4*B3 | 0.8*I3+0.9*I5+0.6*A2+0.4*I4+0.4*B5+0.6*B2+0.2*I2 |
| **C7** | 0.2*I2+0.7*I4+0.7*B1+0.7*A1+0.9*I3+0.5*B3+0.4*B2 | 0.8*I3+0.9*B1+0.6*A2+0.4*I4+0.4*B5+0.6*B2+0.2*I2 |
| **C8** | 0.2*I2+0.7*I4+0.7*B1+0.7*A1+0.9*I3+0.5*A5+0.4*B2 | 0.8*B3+0.9*B1+0.6*A2+0.4*I4+0.4*B5+0.6*B2+0.2*I2 |
|  | **Serie 7** | **Serie 8** |
| **C1** | 0.2*I2+0.1*I3+0.7*A1+0.4*A2+0.6*A3+0.5*A5+0.2*B2 | 1.0*I1+0.9*I2+0.7*I5+0.7*A1+0.5*A2+0.6*A4+0.1*B5 |
| **C2** | 0.2*A4+0.1*I3+0.7*A1+0.4*A2+0.6*A3+0.5*A5+0.2*B2 | 1.0*I1+0.9*I2+0.7*I5+0.7*I4+0.5*A2+0.6*A4+0.1*B5 |
| **C3** | 0.2*A4+0.1*I3+0.7*I4+0.4*A2+0.6*A3+0.5*A5+0.2*B2 | 1.0*I1+0.9*I2+0.7*I5+0.7*I4+0.5*A2+0.6*A4+0.1*B2 |
| **C4** | 0.2*A4+0.1*I3+0.7*I4+0.4*A2+0.6*B4+0.5*A5+0.2*B2 | 1.0*I1+0.9*I2+0.7*B4+0.7*I4+0.5*A2+0.6*A4+0.1*B2 |
| **C5** | 0.2*A4+0.1*I3+0.7*I4+0.4*A2+0.6*B4+0.5*A5+0.2*B5 | 1.0*A5+0.9*I2+0.7*B4+0.7*I4+0.5*A2+0.6*A4+0.1*B2 |
| **C6** | 0.2*A4+0.1*I3+0.7*I4+0.4*A2+0.6*B4+0.5*I5+0.2*B5 | 1.0*A5+0.9*I2+0.7*B4+0.7*I4+0.5*A3+0.6*A4+0.1*B2 |
| **C7** | 0.2*A4+0.1*B1+0.7*I4+0.4*A2+0.6*B4+0.5*I5+0.2*B5 | 1.0*A5+0.9*I2+0.7*B4+0.7*I4+0.5*A3+0.6*B1+0.1*B2 |
| **C8** | 0.2*A4+0.1*B1+0.7*I4+0.4*I1+0.6*B4+0.5*I5+0.2*B5 | 1.0*A5+0.9*I3+0.7*B4+0.7*I4+0.5*A3+0.6*B1+0.1*B2 |
