# Supplementary material for: Rapid Acoustic Survey for Biodiversity Appraisal
Source: PLoS One. 2008 Dec 30;3(12):e4065. doi: 10.1371/journal.pone.0004065 (PMC2605254; doi:10.1371/journal.pone.0004065)
Supplement: Table S4 — Local meteorological conditions during recording sessions in the two Tanzanian lowland coastal forests. Results are given as mean±sd (sample size). (0.03 MB DOC) [file pone.0004065.s012.doc]

**Table S4. Local meteorological conditions during recording sessions in the two Tanzanian lowland coastal forests.**

Results are given as mean ± sd (sample size).

|  | **Kichi Hills** | **Ngumburuni** |
| --- | --- | --- |
| Temperature (°C) | 23.2±2.5 (14) | 26.8±1.1 (15) |
| Relative humidity (%) | 94.7±6.2 (14) | 80.0±5.1 (15) |
| Cloud cover (discrete index varying from 0 to 4) | 2.36±1.34 (14) | 0.53±1.06 (15) |
| Wind force (discrete index varying from 0 to 4) | 1.6±1.3 (14) | 0.4±0.8 (15) |
